# LncRNAs associated with oxidative stress in diabetic wound healing: Regulatory mechanisms and application prospects

**DOI:** 10.7150/thno.85823

**Published:** 2023-06-26

**Authors:** Qinzhi Yang, Dan Fang, Jinxiang Chen, Shaorun Hu, Ni chen, Jun Jiang, Min Zeng, Mao Luo

**Affiliations:** 1 Key Laboratory of Medical Electrophysiology, Ministry of Education, Drug Discovery Research Center, Southwest Medical University, Luzhou, China.; 2 Laboratory for Cardiovascular Pharmacology, Department of Pharmacology, School of Pharmacy, Southwest Medical University, Luzhou, Sichuan, China.; 3 Luzhou Municipal Key Laboratory of Thrombosis and Vascular Biology, Luzhou, Sichuan, China.; 4 Department of General Surgery (Thyroid Surgery), the Affiliated Hospital of Southwest Medical University, Luzhou, Sichuan, China.; 5 Metabolic Vascular Diseases Key Laboratory of Sichuan Province, Luzhou, Sichuan, China.; 6 Department of Pharmacy, the Affiliated Hospital of Southwest Medical University, Luzhou, Sichuan, China.

**Keywords:** diabetes, wound healing, oxidative stress, lncRNAs, therapeutic application

## Abstract

Diabetes is a group of chronic diseases with blood glucose imbalance, and long-term hyperglycaemia causes sustained damage to various organs of the body, resulting in vascular lesions, neuropathy and impaired wound healing. Diabetic wound formation involves a variety of complex mechanisms, and they are characterized by a persistent chronic inflammatory response, degradation of angiogenesis and imbalance of extracellular matrix regulation, all of which are related to oxidative stress. Additionally, repair and healing of diabetic wounds require the participation of a variety of cells, cytokines, genes, and other factors, which together constitute a complex biological regulatory network. Recent studies have shown that long noncoding RNAs (lncRNAs) can be involved in the regulation of several key biological pathways and cellular functions demonstrating their critical role in diabetic wound healing. LncRNAs are a major family of RNAs with limited or no protein-coding function. Numerous studies have recently reported a strong link between oxidative stress and lncRNAs. Given that both lncRNAs and oxidative stress have been identified as potential drivers of diabetic wound healing, their link in diabetic wound healing can be inferred. However, the specific mechanism of oxidative stress related to lncRNAs in diabetic wound healing is still unclear, and elucidating the functions of lncRNAs in these processes remains a major challenge. This article reviews the mechanisms of lncRNAs related to oxidative stress in several stages of diabetic wound healing and discusses diagnostic and treatment potential of lncRNAs to treat diabetic wounds by improving oxidative stress, as well as the challenges of using lncRNAs for this purpose. It is hoped that these results will provide new targets and strategies for the diagnosis and treatment of impaired wound healing in diabetic patients.

## Introduction

**Figure 1:**
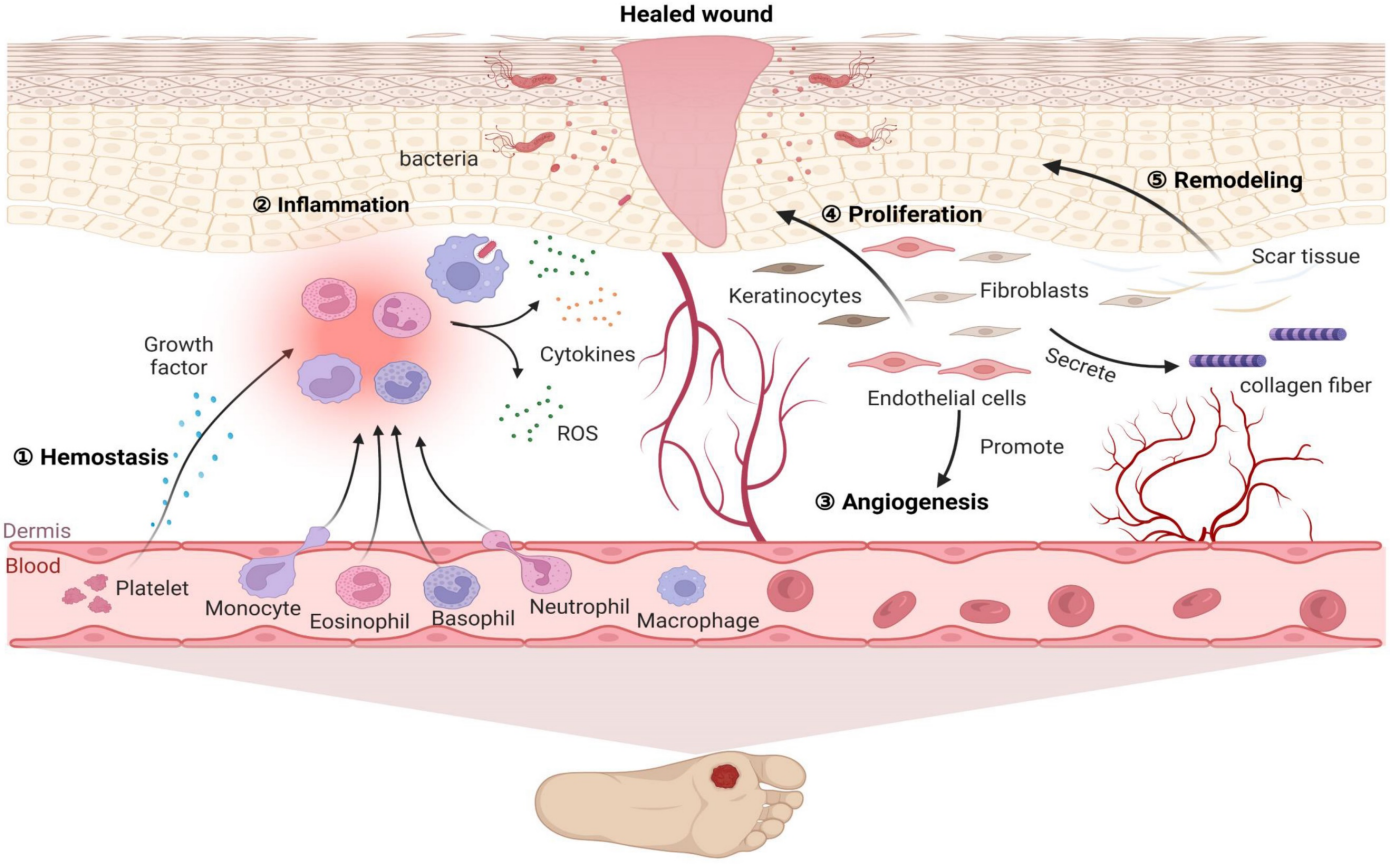
** Pathological characteristics of diabetic wound healing.** Diabetic wounds are characterized by difficulty in hemostasis, chronic inflammation, impaired angiogenesis, slowed cell proliferation and delayed extracellular matrix remodeling. After injury, wounded tissue must establish haemostasis via coagulation and clot formation. Injury is quickly followed by immune cell infiltration and inflammation as a means to clear the wound of damaged tissue and microbes, thus preventing infection and facilitating granulation. Subsequently, fibroblasts, keratinocytes and endothelial cells not only proliferate and migrate to the wounds to promote angiogenesis but also deposit ECM and repopulate the injury site, further facilitating wound closure. Finally, matrix deposition and clearance regulate scar formation. However, for diabetic patients, a long-term high glucose environment promotes high ROS levels in inflammatory cells, causing adhesion of white blood cells to the vascular endothelium and further promoting the inflammation cascade reaction. In turn, the inflammatory reaction stimulates the generation of oxidative stress, thus forming a vicious cycle. Therefore, under the combined action of various factors, these events influence each other and ultimately inhibit diabetic wound repair.

Diabetes mellitus (DM) is a group of metabolic diseases characterized by hyperglycaemia resulting from the interaction of genetic and environmental factors. DM leads to insulin resistance and islet dysfunction and causes high morbidity and disability that pose a serious threat to human health 1. Diabetic cutaneous ulcers (DCU) are caused by long-term poor blood glucose control in diabetic patients, which leads to neuropathy and various degrees of peripheral vascular lesions, resulting in lower limb infection, ulceration, and/or deep tissue destruction 2, 3. DCU is the most common injury leading to lower limb amputation and is one of the serious complications that can result in the death and disability of diabetic patients 4. However, the pathogenesis of DCU has not been fully understood. Hyperglycaemia-induced DCU has been associated with lipid peroxidation, oxidative stress (OS), cell apoptosis and a variety of signal transduction pathways 5, 6. For diabetic patients, a long-term high glucose environment promotes the production of reactive oxygen species, thereby promoting the inflammation cascade reaction and stimulating oxidative stress, thus forming a vicious circle and eventually leading to difficulty healing wounds 6. Additionally, diabetic patients with hyperglycaemia have increased lipid peroxidation-associated oxidative stress and reduced antioxidant capacity, stimulating the formation of advanced glycation end products and autooxidative glycosylation, thus enhancing polyol pathway activity and aggravating destructive processes in the feet of diabetic patients 7. Here, the pathological characteristics of diabetic wounds are shown in **Figure 1**, which shows that a lack of spontaneous neovascularization, bacterial infection of the refractory wound surface, and poorly differentiated dermal cells are the main causes of DCU healing difficulty, and all of these factors are closely related to oxidative stress.

Diabetic wounds characterized by persistent inflammation and the excessive production of reactive oxygen species, thus resulting in impaired wound healing, are unlike typical wounds in that they are slower to heal, causing treatment with conventional topical medications to be a difficult process 8-10. LncRNAs are a class of RNA molecules that do not encode proteins featuring large number and wide variety, with transcripts over 200 nucleotides in length 11. Recently, the abnormal expression of long noncoding RNAs (lncRNAs) in diabetic wounds has received widespread attention since it is an important factor in the development of diabetic wounds 12. Currently, lncRNAs are increasingly recognized as risk factors for oxidative stress and diabetic wound healing. However, the specific regulatory details and mechanisms are still unclear and require further investigation. In this review, we summarize the current findings on lncRNA-mediated gene regulation of oxidative stress and diabetic wound healing. Furthermore, we describe the specific molecular mechanism underlying the regulation of lncRNA-dependent signaling pathways in different cellular processes. These findings may provide a novel theoretical basis and important targets for the clinical application of various diseases including diabetes mellitus and its complication.

## LncRNAs participate in oxidative stress

### Biology research deepens the understanding of lncRNAs and their regulatory role in oxidative stress

**Figure 2:**
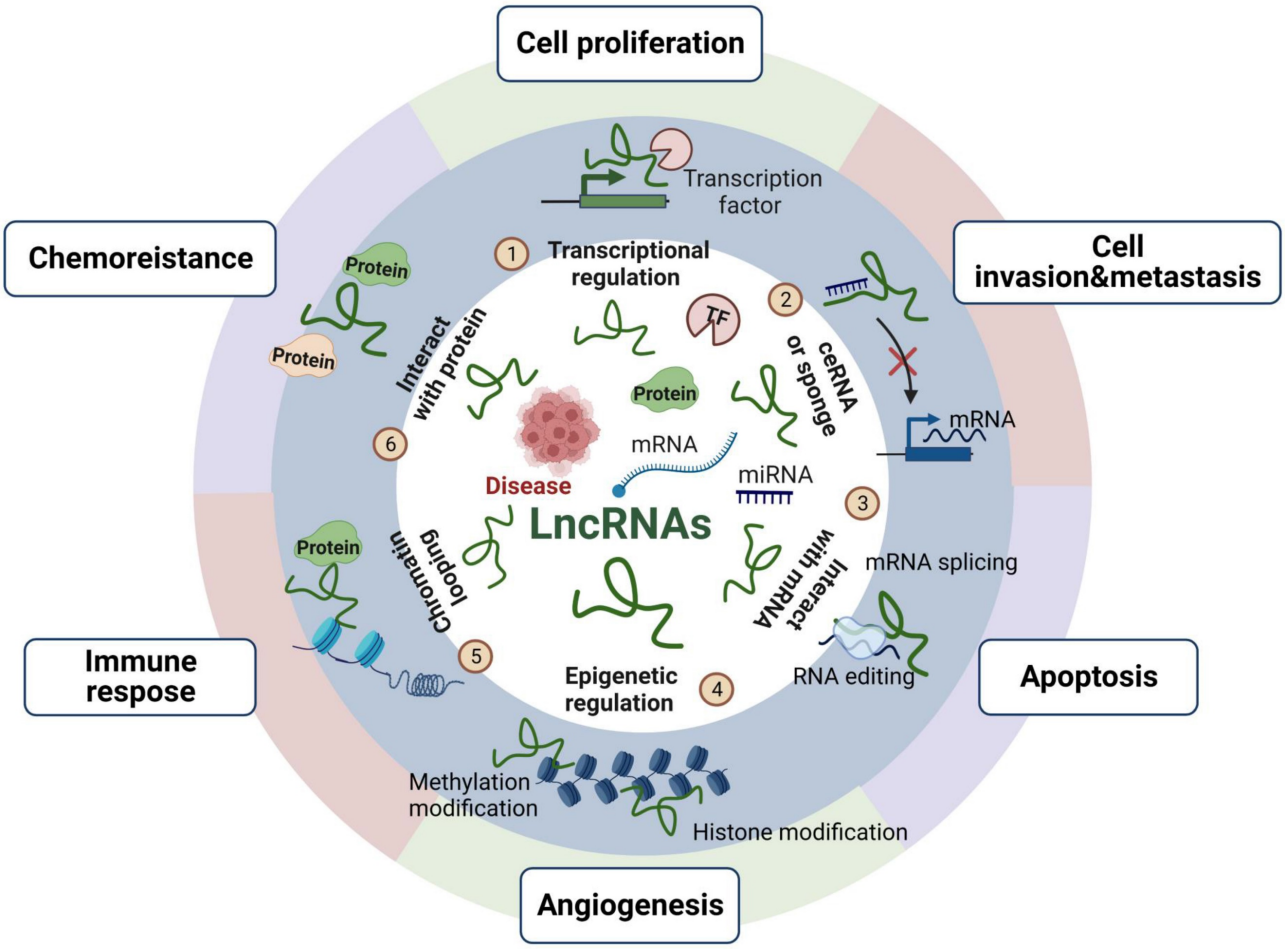
** The mechanisms and functions of lncRNAs.** LncRNAs play roles in cell proliferation, invasion and metastasis, apoptosis, angiogenesis, immune response and chemoresistance mainly through epigenetic modification, transcriptional control and translational regulation. We reported the functions of lncRNAs as follows: ① recruiting transcription factors to activate or repress gene expression; ② acting as competing endogenous RNAs (ceRNAs) or miRNA sponges to regulate miRNA targets and affect their expression; ③ directly binding to mRNA and regulating mRNA stability; ④ regulating epigenetic modifications include histone and DNA methylation, histone acetylation, and ubiquitination; ⑤ promoting chromatin looping; ⑥ interacting with proteins and controlling protein phosphorylation, acetylation, and ubiquitination at the posttranslational level.

LncRNAs, located in the nucleus and cytoplasm of eukaryotic cells, are a class of noncoding RNAs (>200 bp) that interact with different molecules, such as DNA, RNA or proteins, depending on their subcellular distribution, to modulate gene transcription and kinase cascades 13, 14. LncRNAs have cis- or trans-regulation, with cis- regulation acting on adjacent genes and trans-regulation acting on genes far away from their transcription sites 15. Moreover, despite lacking an obvious open reading framework, lncRNAs mainly exert their function via epigenetic modification, transcriptional control and translational regulation, as shown in **Figure 2**, which provides an overview of the mechanisms and functions of lncRNAs. LncRNAs can act as scaffolds to bind a variety of proteins that perform biological functions and can participate in the synthesis and reconstruction of nucleic acid sequences by soliciting or impounding certain protein factors, thereby being involved in a variety of physiological and pathological activities 16. Additionally, lncRNAs can also act as molecular sponges of miRNAs to regulate the biological activities of cells 17.

LncRNAs play key roles in various cell biological processes, such as cell proliferation, differentiation and apoptosis 18. Recent studies have demonstrated the importance of lncRNAs in regulating gene expression in various cells under oxidative stress, including macrophages (MФs), endothelial cells (ECs), and vascular smooth muscle cells (VSMCs). An overview of lncRNAs, their targets in oxidative stress and the roles of oxidative stress-associated lncRNAs in different cells and structures is shown in **Table 1**.

### LncRNAs in immune cells are associated with oxidative stress

The activation of immune cells requires the coordinated action of both catabolic and anabolic pathways 37. To accomplish the pathways, activated immune cells shift towards efficient aerobic glycolysis 38. Early reports have shown that the lncRNA HULC is involved in the regulation of the cellular glycolytic metabolic pathway by interacting with LDHA and PKM2, two key enzymes in the glycolytic pathway 39. Liu J et al. 40 also identified an important molecule of lncRNA Actin Gamma 1 PseudoGene (AGPG) that affects the glycolytic activity and cell proliferation of esophageal cancer by binding the metabolic enzyme PFKFB3. Inefficient glucose utilization reduces ATP production, which is closely related to oxidative damage, indicating that oxidative damage to enzymes caused by the abnormal expression of lncRNAs may be an important reason for the inefficiency of glucose utilization, thus affecting the activity of immune cells. In addition, oxidative damage to mitochondrial DNA induced by lncRNAs may also lead to impaired energy production. For example, studies have shown that deletion of the lncRNA NORAD can lead to genomic instability and mitochondrial dysfunction in mice 41.

Endogenous antioxidant molecules play a central role in the activation of immune cells and inflammatory responses. MФs are indispensable in pathophysiology through their diverse roles in metabolism and the immune response 42. Oxidative phosphorylation (OXPHOS) is inhibited in proinflammatory and activated MФs. The receptors of proinflammatory cytokines such as TNF-α and others on macrophages are coupled to NADPH oxidase (NOX). NOX activation leads to reactive oxygen species (ROS) production within macrophages to counteract the pathogen response or cellular stress response. Previous studies have shown that lncRNAs may be important regulators of TNF-α-mediated oxidative stress in macrophages, suggesting that abnormal expression of lncRNAs can lead to the abnormal expression of NOX regulated by TNF-α, resulting in excessive ROS production. Moreover, Hu et al. 20 found that lncRNA UCA1 sponges miR-206 to aggravate oxidative stress and apoptosis in ox-LDL-induced human macrophages, thus contributing to atherosclerosis. Notably, immune cells are very sensitive to oxidative stress. The high concentration of unsaturated fatty acids in the cell membrane of immune cells and their high sensitivity to peroxidation reactions decreases the immune function of the cells after being stimulated by excessive free radicals, thereby affecting the immune system 43. Overall, abnormally expressed lncRNAs are involved in various functions of immune cells in the body, which may potentially influence the oxidative stress response of immune cells.

### LncRNAs in vascular cells participate in oxidative stress

The two most distinct cell types in the vascular system are endothelial cells (ECs) and mural cells, which include pericytes (PCs) and vascular smooth muscle cells (VSMCs). Interactions of VSMCs, ECs and PCs are important aspects of vascular homeostasis 44. Recent findings have shown that oxidative damage to vascular cells is correlated with lncRNAs. For example, angiotensin II (AngII) is a major inducer of ROS production in VSMCs. Das S et al. 24 found that knockdown of the lncRNA Giver attenuated AngII-induced intracellular ROS production in rat VSMCs (RVSMCs), thus confirming the functional roles of Giver in oxidative stress. In addition, other studies have shown that overexpressed lncRNA Sox2ot aggravated the oxidative stress and inflammation of VSMCs treated with Ang II, and its mechanism is related to the upregulation of early growth response factor-1 (Egr1) expression by competitively binding with miR-145 25.

A substantial amount of evidence supports that lncRNAs play an important role in the oxidative damage of endothelial cells. After overexpression of the lncRNA growth-arrest specific transcript 5 (GAS5), Diao et al. 26 observed decreased GAS5 expression levels in endothelial cells subjected to homocysteine (HCY)-induced cardiac microvascular endothelial cell (CMEC) injury. GAS5 upregulation attenuated HCY-induced CMEC injury by mediating oxidative stress, and its mechanism was related to the upregulation of ABCA1 expression by competitively binding with miR-33a-5p. Similarly, Shen et al. 27 found that lncRNA DLGAP1 antisense RNA 1 (DLGAP1-AS1) silencing inhibits the expression of inflammatory factors, reduces malondialdehyde (MDA) levels and decreases vascular endothelial cell (VEC) apoptosis. The findings suggest that DLGAP1-AS1 silencing can inhibit oxidative stress and exert an antiapoptotic effect. Another study confirmed the involvement of lncRNAs in oxidative damage of ECs induced by NO, and it was shown that the lncRNA uc001pwg.1 could increase endothelial nitric oxide synthase (eNOS) phosphorylation and NO production in vitro 28.

A recent study found that lncRNAs could regulate human pericyte function and were involved in the endoplasmic reticulum (ER) stress response. In 2017, Bischoff FC et al. 29 demonstrated the regulatory feedback role between the ER stress response and HypERlnc expression that affects pericyte function. Endoplasmic reticulum stress-related transcription factors were prominently activated by HypERlnc knockdown, while induction of ER stress significantly lowered HypERlnc levels and induced pericyte dedifferentiation. These observations suggest that lncRNAs may control the occurrence of disease by regulating the level of oxidative stress and reducing endoplasmic reticulum stress.

### LncRNAs in a variety of cells play a role in the regulation of oxidative stress

Several studies have shown that lncRNAs are also involved in the functional regulation of other cells during oxidative stress. For example, the lncRNA OGRU mediates diabetic retinopathy (DR) by competing for miR-320 to promote inflammation and induce the production of ROS by regulating ubiquitin-specific peptidase 14 (USP14) expression in high glucose (HG)-incubated Müller cells 30. Similarly, the suppression of the lncRNA PICSAR reduced IL-6 and IL-8 production in rheumatoid arthritis (RA)-fibroblast-like synoviocytes (FLSs), indicating that PICSAR affects an important function of RA-FLSs 31. In addition, keratinocytes 33, platelets and adipocytes 34, 35 have been revealed to be affected by lncRNA-mediated oxidative stress.

### LncRNAs as regulators of oxidative stress in diabetic wound healing

**Figure 3:**
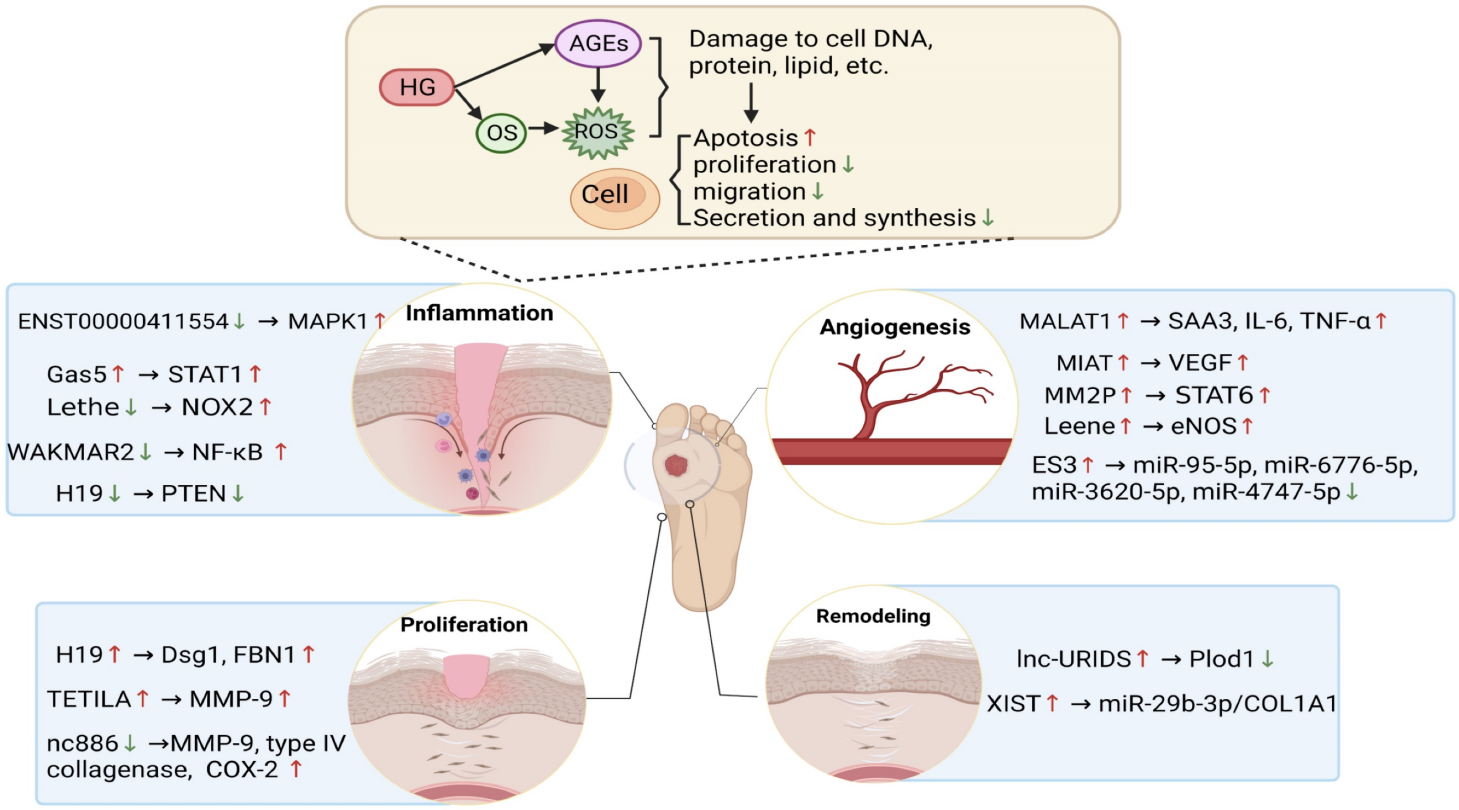
** The biogenesis and regulatory mechanism of lncRNA function is associated with oxidative stress.** The diabetic wound microenvironment, which includes hyperglycaemia, OS and AGEs, induces the abnormal expression of lncRNAs, thereby regulating the expression of downstream genes and oxidative stress levels, further affecting the functions of immune cells, endothelial cells, keratinocytes, and fibroblasts. LncRNAs participate in various stages of diabetic wound healing, such as inflammation, angiogenesis, proliferation and remodelling, to delay or accelerate the repair process of wound healing. Red and blue arrows represent up- and downregulation, respectively.

Wound healing is a complex and orderly biological process that mainly includes five stages: haemostasis, inflammation, angiogenesis, proliferation and remodelling 45. Oxidative stress is one of the complex mechanisms that causes diabetic wound healing difficulties 5. Multiple studies have shown that lncRNAs are involved in various stages of diabetes wound healing and play a critical role in oxidative stress. Thus, it is necessary to understand how the abnormal regulation of expressed lncRNAs delays or accelerates the repair process of wound healing. This part mainly reviews the lncRNAs in each stage of diabetic wound healing and their roles in each phase impacted by oxidative stress, as shown in **Table 2** and **Figure 3**, which exhibit the biogenesis and regulation mechanism of oxidative stress associated lncRNA function and that play a vital role in diabetic wound healing.

#### Haemostasis

Platelets are essential components of hemostasis that provides prompt control of blood loss due to vascular injury 67. Platelets from diabetic patients with poor blood glucose control tend to aggregate 68. Immediately after trauma, haemostasis occurs first, and then platelets aggregate to form fibrin clots via intrinsic mechanisms. This clot formation is followed by platelets releasing large amounts of growth factors, which constrict blood vessels, limit bleeding and attract inflammatory cells to migrate to the wound site and disinfect the wound surface 69. This process is associated with the chemotaxis of many cell types, such as macrophages, white blood cells, vascular smooth muscle cells and the fibroblasts mentioned above, and the dysfunction caused by the oxidative damage of these cells is closely related to lncRNAs. In addition, under diabetic conditions, platelets have been shown to be less responsive to NO released from the vascular endothelium, which normally reduces platelet aggregation after vascular injury, and previous studies have shown that lncRNAs also regulate NO expression in vascular cells 70. Therefore, platelet dysfunction in diabetic patients can not only hinder wound healing by reducing coagulation during haemostasis but also contribute to the development of microvascular disease in diabetic patients. While lncRNAs governing this early phase of diabetic wound healing has not been clearly defined, several lncRNAs implicated in thrombus formation may be involved.

#### Inflammation

Neutrophils are the main cell types involved in the early stages of inflammation 71. In diabetic wounds, a state of sustained high blood sugar can shorten the lifespan and increase the clearance rate of neutrophils, resulting in them not being able to reach the base of the wound in a timely manner. Instead, they scatter around the wound surface and combine with advanced glycation end products outside the vascular tissue to release a large number of inflammatory cytokines, aggravating the inflammatory response and oxidative stress and hindering wound healing 72, 73. In addition, MФs persistently remain in the proinflammatory M1 phenotype in diabetic wounds, which further promotes inflammation and inhibits the initiation of the tissue proliferation period, leading to difficult wound healing 74, 75. A large number of studies have revealed that lncRNAs also play regulatory roles in ROS production and inflammation by controlling the functions of immune cells, including neutrophils, MФ, and immune cells, which are essential in diabetic wound inflammation.

Therefore, the oxidative stress of immune cells caused by lncRNAs through the regulation of ROS production may be an important mechanism underlying diabetic wound inflammation. For example, the existence of immunoregulation‑associated lncRNAs mediated by T lymphocytes in the wound surfaces of diabetic foot ulcers (DFUs) has been demonstrated by Xu et al., who suggested that activation of the MAPK signal transduction pathway mediated by the lncRNA‑ENST00000411554/MAPK1 axis affected the DFU immune regulatory imbalance 46. A number of studies have confirmed that the activation of the MAPK signalling pathway is closely related to ROS and NO synthesis induced by oxides, and the inflammatory response caused by abnormal lncRNA expression in diabetic wound healing may be related to T lymphocyte immune regulation and redox reactions. Additionally, lncRNAs can also affect the inflammatory response by affecting the transformation of macrophage phenotypes. For example, lncRNA-Gas5 promoted the polarization of MФs to the proinflammatory M1 phenotype by inducing the expression of the downstream target gene STAT1 to inhibit wound healing in diabetic patients 47. Importantly, another study demonstrated that the lncRNA Lethe regulates oxidative stress in macrophages cultured under high glucose conditions through the regulation of NOX2 expression, which may affect the inflammatory state in diabetic wounds 48. Specifically, under high glucose conditions, the expression of Lethe is downregulated, resulting in the increased availability of free p65-NFkB to translocate to the nucleus and upregulate NOX2 expression. These results suggest that the abnormal expression of lncRNAs in wounds leads to oxidative stress and immune cell dysfunction, resulting in the abnormal inflammation of diabetic wounds. Although keratinocytes are epidermal cells, they also play an important role in the inflammatory stage of wound healing. Previous studies have shown that the hyperglycaemic environment in diabetic wounds can enhance OS and increase the inflammatory response of keratinocytes, thus stagnating the wound healing process in the inflammatory phase and slowing the healing process 76. Previous studies have shown that the lncRNA wound and keratinocyte migration-associated long noncoding RNA 2 (WAKMAR2) inhibits the production of inflammatory chemokines in keratinocytes by regulating the TGF-b-WAKMAR2-p65-NF-kB signalling axis 49. As previously mentioned, NF-kB is closely related to ROS production. Given their relationship, it can be speculated that lncRNAs are associated with ROS production in keratinocytes at the inflammatory stage. In addition, fibroblasts distributed in the dermis can secrete inflammatory factors and affect wound healing 77. Moreover, the regulation of inflammatory stress by H19 on fibroblasts in diabetic wound healing was validated 32. The results revealed that overexpressed H19 can suppress fibroblast apoptosis and inflammation and stimulate the wound-healing process in mice suffering from DFUs, which involves an interaction with miR-152-3p via the PTEN-mediated PI3K/AKT signalling pathway. A number of studies have shown that the PI3K/AKT pathway is a key signalling pathway that alleviates cellular oxidative stress damage. That is, lncRNAs act on fibroblasts and may regulate oxidative stress-related signalling pathways to regulate wound inflammation.

#### Angiogenesis

Angiogenesis is an essential part of granulation tissue, and another key process in wound healing, mainly involving endothelial cell migration and capillary formation 78. The level of tissue oxygenation is one of the initial microenvironmental cues that activate new blood vessel formation 79. As the injured tissues are cleared, the progression of healing relies upon a new supply of oxygen and nutrients 80. ECs, PCs and VSMCs are closely related to angiogenesis, and the functional impairment of these cells is associated with lncRNAs and oxidative stress. A study reported that the expression of lncRNA MALAT1 was upregulated in the early stage but subsequently downregulated in the late stage under hyperglycaemia, which in turn regulated glucose-induced oxidative stress and the inflammatory response through activation of serum amyloid antigen 3 (SAA3), an inflammatory ligand and target of MALAT1, thus possibly influencing endothelial stability 50. Similarly, the lncRNA myocardial infarction associated transcript (MIAT), a significantly upregulated lncRNA in endothelial cells cultured in high glucose (GH) medium, modulates VEGF levels by sponging miR-150-5p in retinal endothelial cells, thereby promoting angiogenesis 51. Glucose-induced oxidative stress is a key alteration in endothelial cells and is a mediator of most, if not all, other downstream effects 81. Hence, the regulation of MIAT on endothelial angiogenesis under high glucose may also be related to the regulation of oxidative stress. Interestingly, it has been reported recently that MIAT can antagonize the effect of miR-342-3p on inhibiting CASP1 and promote advanced glycation end product-modified bovine serum albumin (AGE-BSA)-induced pericyte pyroptosis 52. AGEs induce pericyte death due to the activation of intracellular oxidative stress and inflammatory responses by binding to the receptor for AGEs (RAGE) on the cell surface 82. Another example is lncRNA-ES3, which triggers the gene silencing of multiple miRNAs by binding to basic helix-loop-helix family member e40 (Bhlhe40), which promotes the HG-induced calcification/senescence of VSMCs 53. With the ageing of VSMCs, vascular atrophy occurs and neovascularization cannot be formed. Notably, the production of high levels of ROS, which is associated with aging, has been demonstrated to be critical for the induction and maintenance of cellular senescence 83. Thus, HG-induced high levels of ROS may have an effect on lncRNA-regulated angiogenesis. Moreover, several lncRNAs have been reported to be involved in macrophage proangiogenic functions. For example, Cao et al. 54 found that lncRNA-MM2P modulates M2 polarization by regulating STAT6 phosphorylation, and MM2P knockout inhibited macrophage-promoted angiogenesis both in vitro and in vivo. Another study showed that M2 macrophage-induced upregulation of prostate cancer-associated transcript 6 (PCAT6) facilitates tumour angiogenesis through modulation of VEGFR2 expression via ceRNA and deubiquitination patterns 84. Overall, manipulation of lncRNAs can regulate the promotion of macrophage-mediated angiogenesis and even improve inflammation, the induced hypoxic environment and abnormal angiogenesis.

Other lncRNAs implicated in angiogenesis and diabetic wound healing include lncRNA-MALAT1, lncRNA-CPS1-IT1 and lncRNA-Leene, which can regulate related cytokines. Jayasuriya R et al. 56 reported a significant reduction in the expression level of lncRNA MALAT1 in DFU patients, which was positively correlated with the expression of angiogenic factors such as nuclear factor erythroid-2-related Factor 2 (Nrf2), hypoxia-inducible factor-1α (HIF-1α) and VEGF. Nrf2 knockout downregulated the expression of HIF-1α and VEGF-A, suggesting that Nrf2 plays a vital role in the regulation of angiogenesis through the MALAT1/HIF-1α loop. In addition, previous studies have found that MALAT1 protects HUVECs against oxidative stress and further promotes angiogenesis by activating the Nrf2 signalling pathway, suggesting that MALAT1 may have a protective effect against vascular endothelial oxidative stress during the angiogenesis stage 85. Another genome-wide analysis of gene expression changes in skin from patients with type 2 diabetes (DM2) found that CPS1-IT1 was significantly downregulated and involved in DM2 57. Moreover, high glucose-induced high oxygen inhibited hypoxia-inducible factor-1α (HIF-1α) expression, which was controlled by CPS-IT1, and inhibited neovascularization 59. Therefore, it is speculated that the management of CPS1-IT1 in the process of diabetic wound healing may occur through HIF-1α, which further promotes angiogenesis through the effect of antioxidative stress. Furthermore, eNOS is one of the key regulators of endothelial homeostasis and vascular function 86. This decoupling not only fails to produce enough NO, but also interferes with the dysfunction of endothelial progenitor cells (EPCs), which interferes with neovascularization and wound healing 87. Studies have shown that an enhancer-associated lncRNA that enhances eNOS expression (LEENE) promotes eNOS transcription, eNOS-derived NO bioavailability, and endothelial function, which inhibits OS-induced antiangiogenesis 60. Collectively, lncRNAs can promote angiogenesis and accelerate wound healing in diabetic wounds by improving oxidative stress and regulating the production of various cytokines involved in initiating and maintaining the healing process.

#### Re-epithelialization (proliferation)

The epidermis is the first barrier against the external environment, and epidermal cells are one of the main repair cells in wound healing 88, 89. Keratinocytes are important cells for the epidermis, and dysregulated lncRNAs mainly regulate wound epithelialization by affecting keratinocyte functions. Cross-talk between fibroblasts and keratinocytes is crucial to stimulate the proliferation of keratinocytes which regenerates epithelium integrity. It was reported that lncRNA H19 directly binds to miR-130b-3p and inhibits keratinocyte differentiation by targeting Dsg1 33. Subsequently, Li et al. 61 constructed a DFU model in mice and found that the expression of miR-29b and its target gene FBN1 involved in wound healing was regulated by H19, which enhances fibroblast proliferation and migration and accelerates epidermal epithelialization of wounds, thus facilitating wound healing through activation of the TGF-β/Smad pathway. Similarly, MMPs play an indispensable role in the migration of keratinocytes. Excess MMP levels can lead to the degradation of the ECM and various growth factors, including TGF-β1 and VEGF, affecting the re-epithelialization and remodelling of wounds 90. Studies have shown that MMP-9 is present in more than 50% of chronic wounds, and its downregulated expression contributes to diabetic wound healing 91, 92.

Recent studies identified a ten-eleven translocation 2 (TET2)-interacting long noncoding RNA (TETILA) that can regulate MMP-9 expression and is upregulated in human diabetic skin tissues. Specifically, TETILA recruits thymine-DNA glycosylase (TDG), which simultaneously interacts with TET2, for base excision repair-mediated MMP-9 promoter demethylation and MMP-9 transcriptional activation, ultimately reducing keratinocyte migration and inhibiting re-epithelialization and wound healing 62. Conversely, lncRNA-nc886 inhibits MMP-9 expression in human keratinocytes 63. In addition, intracellular ROS can be generated and play a considerable role as signalling molecules under hyperglycaemic conditions and can regulate the expression of MMPs 93, 94. Overall, the results suggested that lncRNA interference with keratinocyte function through targeted regulation of MMP may be mediated by ROS produced by high glucose, thus promoting wound re-epithelialization.

#### Remodelling phase

An emerging handful of lncRNAs have been implicated in fibroblasts and participate in the remodeling phase. Hu et al. 64 explored a novel lncRNA, MRAK052872 (named lnc-URIDS), which was upregulated in diabetic skin and dermal fibroblasts. Lnc-URIDS knockdown promoted the migration of dermal fibroblasts treated with advanced glycation end products (AGEs) in vitro and accelerated diabetic wound healing in vivo. Mechanistically, lnc-URIDS targets procollagen-lysine, 2-oxoglutarate 5-dioxygenase 1 (Plod1), a critical enzyme responsible for collagen cross-linking, resulting in the decreased protein stability of Plod1, which leads to the dysregulation of collagen production and deposition and delays wound healing. Another study found that adipose-derived stem cell (ASC)-short-hair interfering RNA PLOD1 (shPLOD1) reduced fibrosis in injured rat regions, likely through the suppression of ROS levels 65. Moreover, ROS are critical for fibrogenesis proliferation and ECM secretion and may affect collagen production and deposition regulated by the binding of lnc-URIDS to Plod1. In addition, lncRNA X-inactive specific transcript (XIST) promoted ECM synthesis and human skin fibroblast (HSF) proliferation and migration by sponging miR-29b-3p and targeting collagen 1 alpha 1 (COL1A1) to promote wound healing 66. In summary, several lncRNAs have been studied as regulators of tissue remodelling, especially under the mediation of oxidative stress. Therefore, further research at all stages of wound healing is needed to identify additional lncRNAs involved in the regulation of wound healing.

### The mechanism of lncRNAs in signalling pathways involved in both oxidative stress and diabetic wound healing

**Figure 4:**
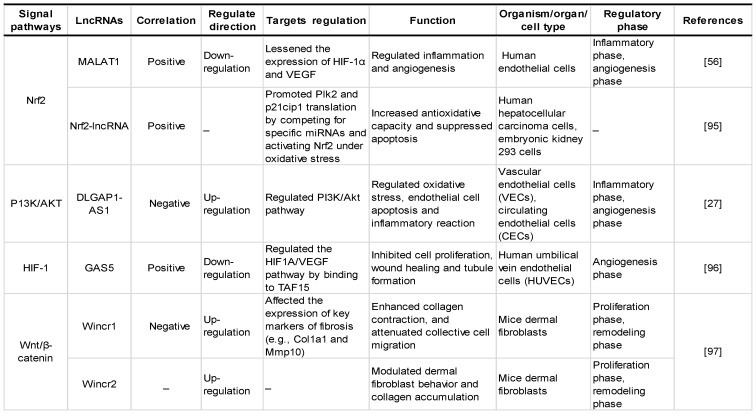

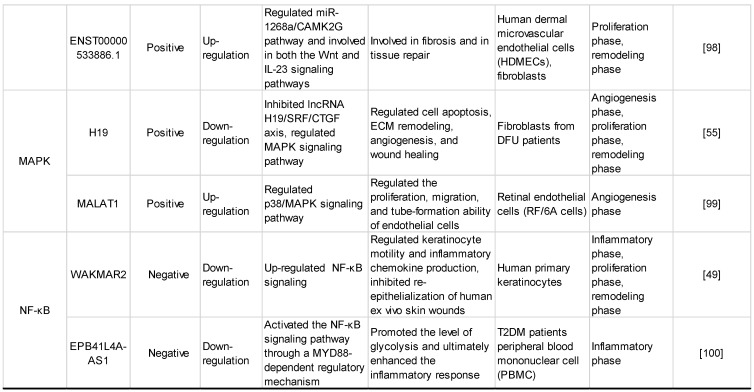

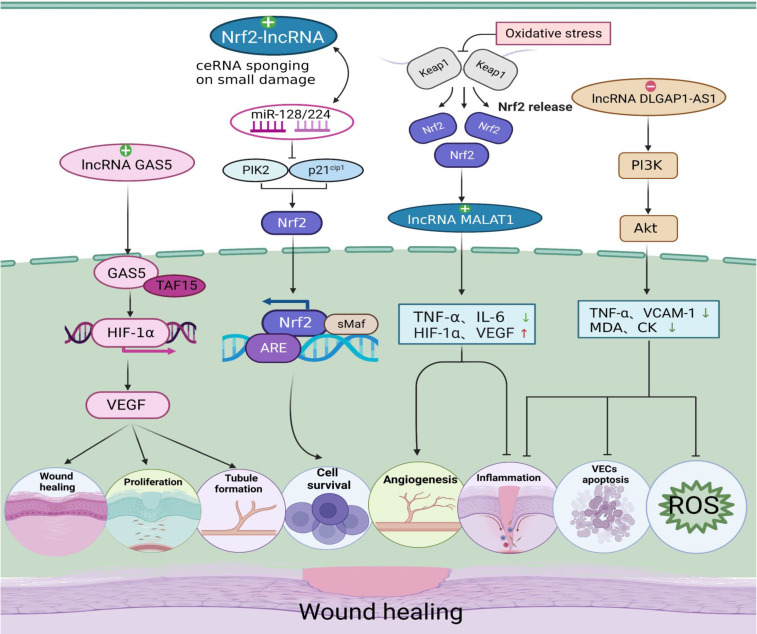
** Regulatory effects of oxidative stress-associated lncRNA pathways on diabetic wound healing.** Nrf2-lncRNA acts as a competing endogenous RNA (ceRNA) sponge for miRNAs 128 and 224. miR-224 and miR-128 inhibit PIK2 and p21cip1, which are involved in p53-dependent stress, thereby enhancing the transcription of Nrf2 and cell survival. LncRNA MALAT1 is regulated by Nrf2, which has a positive correlation with Nrf2 expression in wild-type cells. MALAT1 downregulates the expression of TNF-α and IL-6 to promote angiogenesis and upregulates the expression of HIF-1α and VEGF to reduce inflammation. LncRNA DLGAP1-AS1 silencing activates the PI3K/Akt pathway, causing the levels of TNF-α and VCAM-1 to be decreased and the MDA levels and CK activity to be decreased. DLGAP1-AS1 silencing suppresses oxidative stress, endothelial cell apoptosis and inflammatory reactions. LncRNA GAS5 activates the HIF1A/VEGF pathway by binding to TAF15, and promotes cell proliferation, wound healing and tubule formation via the HIF1A/VEGF pathway in HUVECs. Therefore, when lncRNAs activate Nrf2, PI3K/AKT, HIF-1, and other signalling pathways, they can regulate the inflammatory response, cell proliferation, migration, and angiogenesis during the wound healing process, thus promoting wound healing.

Chronic hyperglycaemia can activate various signalling pathways in the mitochondrial reactive oxygen species system to activate and regulate various transcription factors and induce gene transcription and the expression of related functional proteins, which in turn participate in cell proliferation and migration, ultimately affecting the process of diabetic wound healing. Studies have found that lncRNAs regulate oxidative stress-related signalling pathways such as Nrf2, P13K/Akt and HIF-1, which are closely related to diabetic wound healing, as shown in **Table 3** and **Figure 4**, which exhibit the regulatory mechanisms of oxidative stress-associated lncRNAs associated with key genes and signal pathways in diabetic wound healing.

#### Nrf2 signalling pathway

As a redox-sensitive transcription factor, Nrf2 eliminates excessive ROS by upregulating antioxidant response elements, thus regulating ROS levels in organisms 101. Li et al. 102 demonstrated that Nrf2 overexpression increased the protective effect of adipose-derived stem cells (ADSCs) on EPC proliferation and angiogenesis in a high glucose environment and was beneficial to wound healing, during which inflammation and oxidative stress-related protein levels were reduced. More importantly, in the angiogenesis stage of chronic wound healing, lncRNA MALAT1 positively regulates Nrf2 and may protect HUVEC from oxidative damage by activating Nrf2 signaling pathway, which is beneficial to wound healing 56. Separately, another study identified a novel lncRNA named Nrf2-activating lncRNA (Nrf2-lncRNA) transcribed from an upstream region of the microRNA 122 gene (MIR122), which controls cell fate by modulating p53-dependent Nrf2 activation as a miRNA sponge for polo-like kinase (Plk)2 and cyclin-dependent kinase inhibitor 1 (p21^cip1^) 95. Although this study did not demonstrate a direct causal relationship between Nrf2-lncRNA and diabetic wound healing, it may contribute to further research on the antioxidant capacity of lncRNAs and their regulation of diabetic wound healing under oxidative stress conditions.

#### P13K/AKT signalling pathway

Following the activation of PI3K, a cascade of AKT phosphorylation and the second messenger phosphatidylinositol 3 phosphate (GSK-3β) inhibits oxidative damage 103. Yu et al. 104 demonstrated that hyperglycemia-impaired PI3K-Akt signaling may lead to migration, proliferation and angiogenesis dysfunction of endothelial cells in diabetes patients, which is likely to cause slow healing of diabetic wounds. Additionally, Nishikai-Yan Shen T et al. 105 found that IL-6 promoted the migration of nondiabetic fibroblasts during wound healing through the PI3K/Akt signalling pathway, which was dysfunctional in diabetic wounds. The proliferation and migration of fibroblasts can activate collagen production and promote the growth of new granulation, which is an important part of wound healing, further confirming the key role of the PI3K/Akt pathway in diabetic wound healing. Moreover, lncRNA DLGAP1-AS1 activates the phosphoinositol 3-kinase (PI3K)/Akt pathway to control oxidative stress, and endothelial cell apoptosis has also been previously mentioned 27. Hence, lncRNA protects against oxidative damage to endothelial cells induced by high glucose and promotes fibroblast migration through the P13K/AKT signalling pathway, which may be another possible mechanism of lncRNAs in diabetic wound healing.

#### HIF-1 signalling pathway

HIF-1 is an important transcription factor that maintains oxygen homeostasis in hypoxic cells 106. Once the two subunits of HIF-1 (HIF-1α and HIF-1β) are recognized and bound, they can activate the transcription of downstream target genes 107. In the case of hypoxia, the function of HIF-1α is activated and can promote the activation of NADH oxidase (NOX), resulting in ROS overproduction. The level of this enzyme will increase during oxidative stress and eventually exacerbate the oxidative environment 108. Recently, it has been reported that HIF-1α can promote wound healing. β-N-Oxalyl-L-α, β-diaminopropionic acid (L-ODAP) is a natural nonprotein amino acid that can be metabolized in the human body and is also a safe wound healing agent. Sharma et al. 109 conducted an in vitro experiment on the wound healing activity of L-ODAP and confirmed that L-ODAP can upregulate the expression of VEGF, matrix metalloproteinase-2 (MMP-2) and MMP-9 by increasing HIF-1α levels, thus accelerating wound healing. Noncoding gene lncRNAs are also involved in the regulation of the HIF-1 signalling pathway and the healing of chronic skin ulcer wounds. For example, Peng et al. 96 found that in the skin tissues of DFU patients, lncRNA-GAS5 and HIF-1α were downregulated, and GAS5 induced HIF-1α expression by interacting with TAF15. High GAS5 expression positively regulates the HIF-1α/VEGF pathway, which promotes the proliferation of HUVECs and accelerates wound repair and healing.

#### Other signalling pathways

In addition to the above main signaling pathways, several lncRNAs are reported to be involved in the regulation of the Wnt/β-catenin signalling pathway in wound healing, including lncRNA-Wincr1, -Wincr2, and -ENST00000533886.1 97, 98. Moreover, several lncRNAs are involved in the regulation of the MAPK signalling pathway, including lncRNA-H19 and lncRNA-MALAT1 55, 99. Additionally, other lncRNAs are involved in the regulation of the NF-κB pathway, including WAKMAR2 and EPB41L4A-AS1 49, 100. However, to date, few studies have reported that lncRNAs participate both in oxidative stress and diabetic wound repair. Further evidence is needed to establish the specific mechanisms of lncRNAs in other signaling pathways related to oxidative stress.

## Progress and Prospects of lncRNAs in diagnosis and treatment

### LncRNA as a diagnostic biomarker

Deregulated lncRNA expression is found in diabetic patients and serves as a diagnostic biomarker to predict diabetes and its complications, including diabetic wounds. For example, Li X et al. 110 showed that the expression levels of 3 lncRNAs in the peripheral blood increased gradually from the control group to the prediabetes group to the T2DM group, especially lncRNA ENST00000550337.1, suggesting that ENST00000550337.1 may potentially distinguish the severity of diabetes and act as a novel reference index for clinical classification. Another study also found that the expression of GAS5 in the wound surface of patients with diabetes was significantly higher and mainly located in the M1 macrophages, which may be a biomarker for diabetic wounds in the early inflammation phase 47. In addition, the significant increase in the expression level of lncRNA URIDS in the skin and serum samples of DFU patients mentioned above suggests that it may be the key to delayed diabetic wound healing, further providing a basis for diabetic ulcer diagnosis 64. Overall, we draw a speculation that, lncRNAs isolated from body fluids or tissues may be adopted as an accurate biomarker for the clinical evaluation of the severity of diabetic wounds.

### LncRNAs as targets for wound-repair treatment

**Figure 5:**
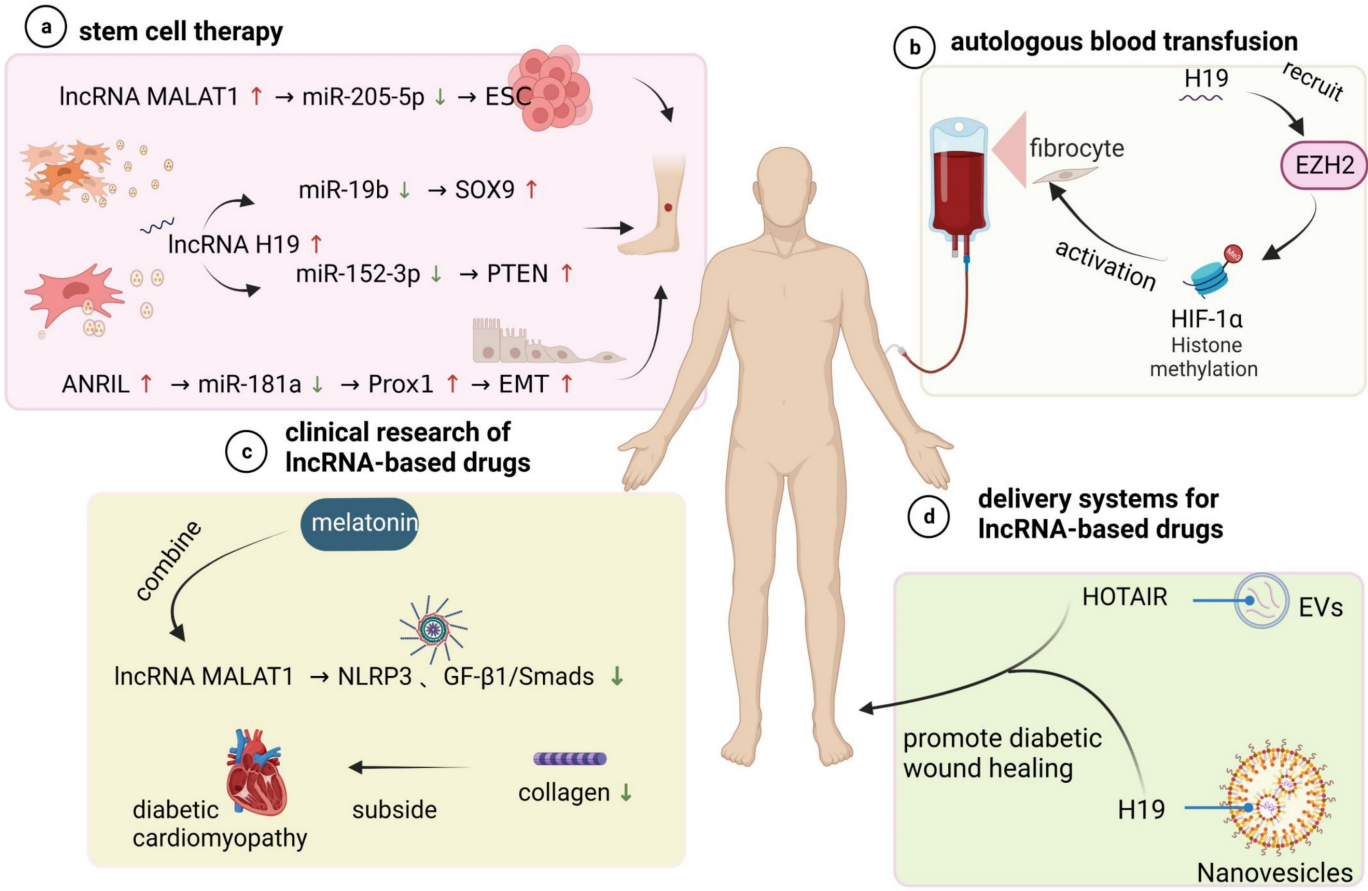
** Prospective application of lncRNAs in the treatment of diabetic wounds. (a) Stem cell therapy promotes diabetic wound healing.** LncRNA MALAT1 acts as a sponge for miR-205-5p to promote the repairing effect of epidermal stem cells (ESCs) on DFUs. Exosomes secreted from different sources (ADSCs) (MSCs) contain lncRNA H19 to accelerate wound healing through two different pathways. lncRNA ANRIL is involved in the induction of tissue epithelial-mesenchymal transition (EMT). (b) Autologous blood transfusion. H19 accelerates fibroblast activation by recruiting ezh2-mediated histone methylation and regulating the HIF-1α signalling pathway, thereby enhancing autologous blood transfusion and promoting postoperative wound healing in diabetic mice. (c) Clinical studies of lncRNA-based drugs. Most drugs have potential lncRNA binding sites. Melatonin attenuates diabetic cardiomyopathy (DCM) by inhibiting lncRNA malat1-mediated NLRP3 inflammatory vesicles and the TGF-β1/Smad signalling pathway. (d) LncRNA drug delivery system. The use of different nanoparticles and extracellular vesicles (EVs) as carriers of lncRNA complexes for exogenous supplementation or inhibition of related lncRNAs to restore normal expression levels and accelerate diabetic wound healing.

LncRNAs play a non-negligible role in the regulation of diabetic wounds and can, therefore, be used as alternative therapeutic targets. Many lncRNAs have been applied to advanced treatment methods in different forms, along with many relevant experiments in vi*tro* and* in vivo*, as shown in **Figure 5**, which provides drugs directly or indirectly regulating lncRNAs to promote the healing of diabetic wound healing.

#### Stem cell therapy

Stem cell transplantation can directly promote local cell wound repair and skin wound healing 111. In diabetic wounds, stem cells not only differentiate into specific cells to replace dysfunctional cells and repair damaged tissue but also promote the migration of fibroblasts, the thickening of granulation tissue, and the secretion of various cytokines to promote angiogenesis 112-117. Interestingly, studies have demonstrated that lncRNAs play an important role in stem cell therapy of diabetic wound healing. For instance, lncRNA MALAT1 functions as a sponge RNA for miR-205-5p to increase therapeutic effects of epidermal stem cells (ESCs) on DF, which was shown to be associated with improved vascularization on the disease site 118. In another study, it was shown that the high expression of lncRNA H19 in exosomes derived from adipose mesenchymal stem cells (ADSCs) may upregulate SOX9 expression via miR-19b to accelerate the wound healing of skin tissues 119. The role of mesenchymal stem cell (MSC)-derived exosomal H19 in promoting DFU wound healing was also proven in a similar study 32. These studies suggest that lncRNAs may function as promising adjunctive agents to enhance stem cell therapy for DFU and provide support for future clinical trials of stem cell therapy.

In addition, other lncRNAs are reported to be involved in tissue epithelial-mesenchymal transition (EMT) formation, which may serve as a repair system in vivo to renew cells and achieve tissue regeneration and homeostasis. For example, He et al. 120 showed that long noncoding RNA-antisense noncoding RNA in the INK4 locus (ANRIL) could sponge miR-181a to enhance Prox1 expression and rescue the impairments of EMT formation of lymphatic endothelial cells (LECs) caused by HG. Therefore, regulating EMT formation by using lncRNAs to promote wound healing is another feasible method that may provide insights for the design of new therapies to improve wound healing efficacy in diabetes.

#### Autologous blood transfusion

Autologous blood transfusion (ABT) is the collection and reinfusion or transfusion of one's own blood or blood components before, during or after surgical procedures and may be another promising direction in lncRNA-based therapy 121. Clinical treatment with ABT can prevent further damage to red blood cell function in diabetic patients and thus improve the function of fibroblasts function, which play a role in wound healing 122. Apart from these findings, the function of lncRNAs in wound healing impaired by DM through modified preservative fluid-preserved ABT was demonstrated. Guo et al. 123 indicated that modified preservative fluid-preserved autologous blood upregulated lncRNA H19 expression in fibroblasts and maintained better oxygen-carrying and oxygen release capacities as well as coagulation function. Correspondingly, H19 upregulation accelerated fibroblast activation by recruiting EZH2-mediated histone methylation and modulating the HIF-1α signalling pathway, thereby augmenting the process of modified preservative fluid-preserved autologous blood and enhancing postoperative wound healing in diabetic mice.

### Clinical Advancements of lncRNA-Based Drugs

It is well established that lncRNAs may affect normal gene expression and disease progression, and most drugs currently have potential lncRNA binding sites. This notion provides a basis for the development of drugs that use lncRNAs as therapeutic targets. For example, Che et al. 124 reported that melatonin alleviates diabetic cardiomyopathy (DCM) by inhibiting MALAT1-mediated NLRP3 inflammasome and TGF-β1/Smad signalling. In related experiments, it reduced collagen production and was beneficial to HG-treated cardiac fibroblasts (CFs). In addition, similar effects on collagen and fibroblasts were consistently observed in diabetic wounds, which provides ideas for developing wound healing drugs.

#### Delivery Systems for lncRNA-Based Drugs

Since lncRNAs are abnormally expressed diabetic skin ulcer patients, exogenous supplementation or inhibition of the related lncRNAs to restore the expression levels may be an ideal method to speed the healing of diabetic wounds. Different nanoparticles are currently being investigated as vehicles to deliver lncRNA modulation complexes to specifically targeted cells and tissues in an effective, noncytotoxic manner. The use of extracellular vesicles (EVs), nanoscale lipid membrane-bound vesicles that are secreted by cells of both prokaryotes and eukaryotes and carry bioactive cargos, including proteins, nucleic acids and lipids from source cells, may be an promising approach well suited to wound repair 125. For example, Born L J et al. 126 examined the therapeutic potential of EVs from mesenchymal stem/stromal cells (MSCs) transfected to overexpress long noncoding RNA HOX transcript antisense RNA (HOTAIR). The results showed that MSC EVs containing significantly increased levels of HOTAIR were effective in promoting wound bed vascularization and enhancing wound repair in diabetic mice, which supports the findings of the small number of other studies on EV-mediated lncRNA therapeutic effects. Similarly, Tao et al. 127 delivered lncRNA-H19 to wounds using extracellular vesicle-mimetic nanovesicles (EMNVs) as an effective nanodrug delivery system and observed significant stimulation of angiogenesis. These recent advances in EV-related research have provided feasible approaches for developing emerging therapeutic nanoplatforms using EVs.

## Conclusions and perspective

The common factors that promote diabetic wound healing are increased fibroblast proliferation, enhanced angiogenesis, the accelerated rate of regenerative epithelialization, and decreased apoptosis. Among them, ROS levels increased by ROS generators or decreased by antioxidant systems are recognized by ROS sensors that trigger signals that affect lncRNAs, which in turn influence ROS generators or antioxidant systems, thereby affecting key cellular functions. Considering the link between lncRNAs and oxidative stress, their common potential driving role in diabetic wound healing can be inferred. Therefore, in terms of mechanism, lncRNAs may regulate gene expression of immune cells, endothelial cells, vascular smooth muscle cells, pericytes and other cells through oxidative stress, thus affecting cell function. In addition, a variety of oxidative stress signalling pathways and influencing factors are involved in the regulation of lncRNAs in diabetic wound healing, including Nrf2, P13K/Akt, HIF-1, Wnt/β-catenin, MAPK, and NF-κB. The participation of a variety of cells, cytokines, ROS, genes, and other factors, which together constitute a complex biological network of regulation, and ultimately affect the repair and healing of diabetic wounds.

In recent years, the traditional treatment methods for diabetic wounds mostly use debridement, drainage, dressing and other treatments on the basis of blood glucose control and circulation improvement, which may have poor curative effects. Moreover, long-term treatment of the disease may allow the drug to accumulate in the body, leading to increased adverse reactions and increased economic burden. It is critical to find safe and effective treatment for the repair and healing of diabetic wounds. The application of non-coding RNAs such as the lncRNAs presents an attractive breakthrough point for the treatment of diabetic wound healing, and treatment strategies based on lncRNAs have unique advantages as the lncRNAs constitute a powerful class of gene regulators. Usually, the same lncRNA gene is associated with multiple target genes, and one target gene is regulated by multiple lncRNAs. As a result, targeted therapy may exhibit a higher therapeutic efficacy, making the development of new drugs and new therapies for diabetic wounds more promising. For example, nanocomplexes can effectively deliver lncRNAs with optimal targeting specificity. LncRNA loaded on oxygen-deficient molybdenum-based nanodots (MoO_3- X_) to prepare nanocomplexes may be an emerging therapeutic strategy, thus improving the ROS scavenging effect and bactericidal performance of MoO_3- X_ nanodots and ultimately accelerating wound healing 128. Notably, MoO_3- X_ nanodots have a singular composition and are simple to prepare, which is more conducive to their clinical transformation. Similarly, Zhao et al. 129 developed a ROS-scavenging hydrogel loaded with different therapeutic contents for effective wound regeneration under various complex conditions. Accordingly, it is speculated that ROS-scavenging hydrogels containing lncRNA-binding site drugs have greater efficacy in treating difficult-to-heal wounds, such as infected diabetic wounds. In addition to controlling the expression level of lncRNAs and ROS level in wounds through the delivery system, damage resulting from abnormal lncRNA expression and oxidative stress can also be reversed by directly using lncRNA mimics or inhibitors. As a potentially important class of therapeutic molecules, lncRNA mimics and inhibitors can be extensively modified, which can promote their high stability in vivo and reduce immunogenicity, as well as be easily labelled with organ-targeted peptides for tissue-specific distribution. Moreover, lncRNAs can be applied in different forms to advanced treatment methods, including stem cell therapy and autologous blood transfusion, to improve the healing of diabetic wounds.

Overall, it is expected that the role of lncRNAs in diabetic wound healing under oxidative stress will be useful to confirm or even discover novel potential targets and cellular pathways, which not only provides accurate diagnostic biomarkers for the clinical evaluation of the severity of diabetic wounds but also provides new insights for the treatment of diabetic wounds. However, the current research on lncRNAs has just started, still facing some of the following challenges, hence further research into their clinical application is needed. First, use of lncRNAs as diagnostic markers due to their instability, low sensitivity, and individual differences further limits application as therapeutic targets. LncRNAs may participate in several important processes of wound healing; however, their roles, expressions and modes of action may differ at each stage. In addition, differences in the level of inflammation in each patient will also lead to differences in the activities of lncRNAs, giving it limited specificity. On the other hand, in addition to dynamic and individual differences, the interactions between lncRNAs are complex. As mentioned above, lncRNAs can have multiple downstream target genes, regulate different signaling pathways, and participate in different physiological processes of various cells and tissues, so their actions differ across cells, tissues, and target sites. In addition, the use of lncRNAs in disease diagnosis and treatment is based on changes in their expression levels or restoration of expression through artificial supplementation for therapeutic purposes, requiring more information about patients. At the same time, due to the individual differences of lncRNAs and patients, the question of whether reversing expression levels will cause serious side effects must also be taken into account. Therefore, the consideration of the above issues further increase the challenge of clinical application of lncRNAs. Besides, the translation of all in vitro, in silico, and animal model discoveries to the clinical treatment of human diabetes is also a huge challenge. Specifically, most of these studies are mainly basic research; hence, preclinical and clinical studies are required to verify clinical applications. Furthermore, the transformation of lncRNAs into drugs is another difficulty. We need not only reliable gene-modification technology but also more efficient drug delivery systems to ensure specific targeting and accurate transmission of target drugs. In a word, a deeper understanding of lncRNA-dependent oxidative stress responses involved in diabetic wound is needed to screen for more specific lncRNAs. This will open up a wide range of biomarkers and therapeutic targets in new areas, to the benefit of numerous patients with diabetic wound healing disorder.

## Figures and Tables

**Table 1:**
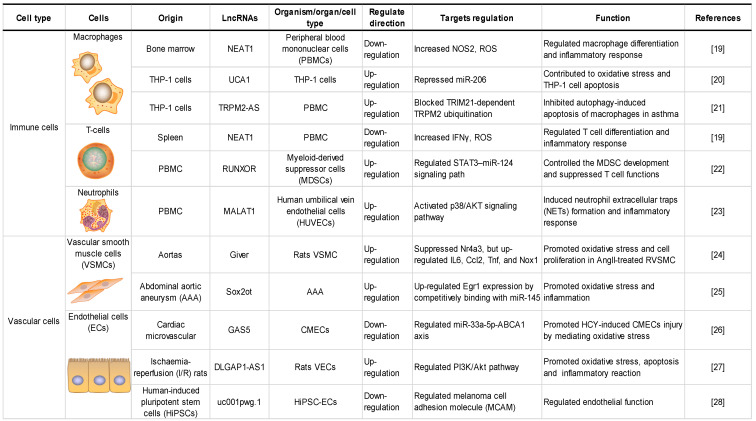

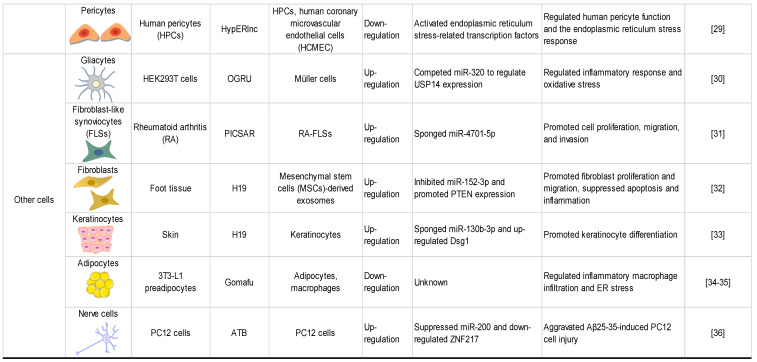
Roles of oxidative stress associated lncRNAs in different cells and structures.

**Table 2:**
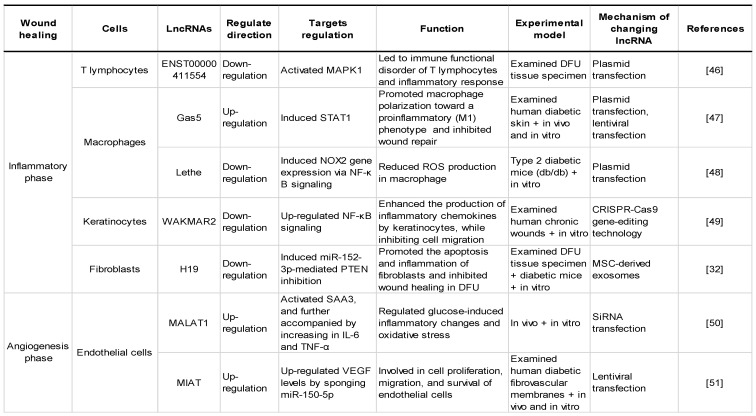

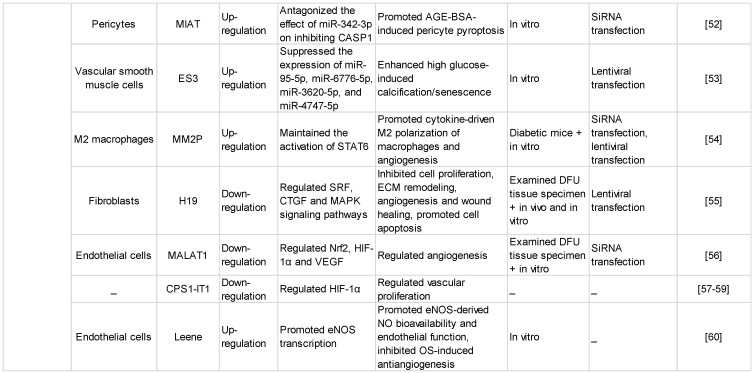

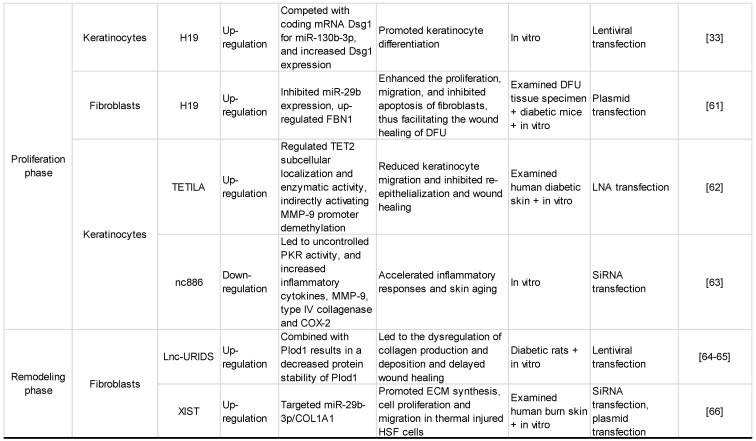
Regulatory effects of certain common oxidative stress-associated lncRNAs on the pathology of diabetic wounds.

**Table 3:** Regulatory effects of oxidative stress-associated lncRNA pathways on diabetic wound healing.

## Abbreviations

LncRNAs: long noncoding RNAs
DM: diabetes mellitus
DCU: diabetic cutaneous ulcers
OS: oxidative stress
MФ: macrophages
ECs: endothelial cells
VSMCs: vascular smooth muscle cells
AGPG: actin gamma 1 pseudogene
OXPHOS: oxidative phosphorylation
NOX: NADPH oxidase
ROS: reactive oxygen species
PCs: pericytes
AngII: Angiotensin II
RVSMC: rat VSMC
Egr1: early growth response factor-1
GAS5: growth-arrest specific transcript 5
HCY: homocysteine
CMECs: cardiac microvascular endothelial cells
DLGAP1-AS1: DLGAP1 antisense RNA 1
MDA: malondialdehyde
eNOS: endothelial nitric oxide synthase
ER: endoplasmic reticulum
DR: diabetic retinopathy
USP14: ubiquitin-specific peptidase 14
HG: high glucose
RA: rheumatoid arthritis
FLSs: Fibroblast-like synoviocytes
DFUs: diabetic foot ulcers
WAKMAR2: wound and keratinocyte migration-associated long noncoding RNA 2
SAA3: serum amyloid antigen 3
MIAT: myocardial infarction associated transcript
AGE-BSA: advanced glycation end product modified bovine serum albumin
RAGE: receptor for AGEs
Bhlhe40: basic helix-loop-helix family member e40
PCAT6: prostate cancer-associated transcript 6
Nrf2: nuclear factor erythroid-2-related factor 2
HIF-1α: hypoxia-inducible factor-1α
DM2: type 2 diabetes
EPCs: endothelial progenitor cells
LEENE: enhances eNOS expression
TET2: ten-eleven translocation 2
TETILA: ten-eleven translocation 2-interacting long noncoding RNA
TDG: thymine-DNA glycosylase
AGEs: advanced glycation end products
Plod1: procollagen-lysine 2-oxoglutarate 5-dioxygenase 1
ASCs: adipose-derived stem cells
shPLOD1: short-hair interfering RNA PLOD1
XIST: X-inactive specific transcript
HSF: human skin fibroblasts
COL1A1: collagen 1 alpha 1
ADSCs: adipose-derived stem cells
Nrf2-lncRNA: Nrf2-activating lncRNA
MIR122: microRNA 122 gene
Plk: polo-like kinase
p21cip1: cyclin-dependent kinase inhibitor 1
GSK-3β: phosphatidylinositol 3 phosphate
PI3K: phosphoinositol 3-kinase
NOX: NADH oxidase
L-ODAP, β-N-oxalyl-L-α: β-diaminopropionic acid
MMP-2: matrix metalloproteinase-2
ESCs: epidermal stem cells
ADSCs: adipose mesenchymal stem cells
MSCs: mesenchymal stem/stroma cells
EMT: epithelial-mesenchymal transition
ANRIL: antisense noncoding RNA in the INK4 locus
LECs: lymphatic endothelial cells
ABT: autologous blood transfusion
DCM: diabetic cardiomyopathy
CFs: cardiac fibroblasts
EVs: extracellular vesicles
HOTAIR: HOX transcript antisense RNA
EMNVs: extracellular vesicle-mimetic nanovesicles
MoO_3- X_: molybdenum-based nanodots
